# Folic acid effect on homocysteine, sortilin levels and glycemic control in type 2 diabetes mellitus patients

**DOI:** 10.1038/s41387-022-00210-6

**Published:** 2022-06-22

**Authors:** Noha M. El-khodary, Hossam Dabees, Rehab H. Werida

**Affiliations:** 1 Clinical Pharmacy Department, Faculty of Pharmacy, Kafrelsheikh University, Kafrelsheikh City, Egypt; 2 Internal Medicine and Diabetes Department, Damanhour Medical National Institute, Damanhour City, Egypt; 3 Clinical Pharmacy & Pharmacy Practice Department, Faculty of Pharmacy, Damanhour University, Damanhour City, Egypt

**Keywords:** Endocrine system and metabolic diseases, Lipids

## Abstract

**Aim:**

The present study aimed to determine the folic acid supplement (FAS) effects on serum homocysteine and sortilin levels, glycemic indices, and lipid profile in type II diabetic patients.

**Method:**

A double-blind randomized controlled clinical trial have been performed on 100 patients with T2DM randomly divided into two groups that received either placebo or folic acid 5 mg/d for 12 weeks.

**Results:**

FAS caused a significant decrease in homocysteine and sortilin serum levels (28.2% and 33.7%, *P* < 0.0001, respectively). After 3 months of intervention, 8.7% decrease in fasting blood glucose (*P* = 0.0005), 8.2% in HbA1c (*P* = 0.0002), 13.7% in serum insulin (*P* < 0.0001) and 21.7% in insulin resistance (*P* < 0.0001) were found in the folic acid group, however no significant difference was observed in the placebo group. Serum hs-CRP level showed significant positive associations with sortilin (*r* = 0.237, *P* = 0.018), homocysteine (*r* = 0.308, *P* = 0.002) and fasting blood glucose (*r* = 0.342, *P* = 0.000). There were no significant changes in lipid profile in both groups after 12 weeks.

**Conclusion:**

FAS might be beneficial for reducing homocysteine and sortilin levels, enhancing glycemic control, and improved insulin resistance in patients with T2DM.

## Introduction

Diabetes mellitus is one of the extremely common chronic ailments in nearly all countries and major health care problem worldwide [1].

Multiple risk factors have been recognized as potential contributors for cardiovascular disease (CVD) in diabetic patients including hyperglycemia, elevated glycated hemoglobin (HbA1c) concentrations, hyperinsulinemia, hypertension, obesity, hypercholesterolemia, hypertriglyceridemia, and smoking [2].

In addition to these factors, it is known that elevated homocysteine (Hcy) levels can be associated with cardiovascular disease and increased atherosclerosis risk, peripheral vascular disease, ischemic heart disease, and stroke [3–5].

In diabetic patients, hyperhomocysteinemia is correlated with insulin resistance [6], dyslipidemia, and poor control of the disease [7, 8]. Hyperhomocysteinemia results in the worsening of T2DM by induction of reversible β-islet cell dysfunction and insulin secretion inhibition [9]. Levels of circulating Hcy are raised in patients with T2DM in contrast to non-diabetic individuals [10].

Folic acid is an essential factor in determining plasma Hcy concentrations [11, 12]. Therefore, folic acid supplementation (FAS) lowers the Hcy level by increasing the 5-methyltetrahydrofolate intracellular pool, which may improve the overall management of diabetic patients and may decrease cardiovascular events [3, 10]. Hcy level may be a useful predictor of end-organ damage, including cardiac, carotid, and renal diseases, in newly diagnosed T2DM patients [13].

Insulin resistance, β-cell dysfunction, poor glucose tolerance, and mitochondrial dysfunction have all been linked to oxidative stress [14]. Because of its antioxidant capabilities, the folic acid treatment improved the glycemic profile significantly [15]. Inflammation is linked to the development of insulin resistance. Proinflammatory cytokines including tumor necrosis factor-alpha (TNF-α) and IL-6 have been shown to affect insulin signaling, which can lead to insulin resistance [16]. FAS has also been shown to reduce NF-kB activity, which inhibits the proinflammatory process [17].

Chronic inflammatory biomarkers, like C-reactive protein (CRP) levels, have been linked with the existence and extent of the metabolic syndrome [18], preclinical atherosclerosis, and the development of atherosclerosis [19]. Furthermore, it has been reported that high sensitivity C-reactive protein (hs-CRP) is recognized as the predictor of a cardiovascular event [20].

Sortilin is mainly found within cells in sites such as the cell membrane and systemic circulation, and it is also found in the trans-Golgi network [21, 22]. It plays a key role in many biological processes including glucose and lipid metabolism, and atherosclerosis which are risk factors for CVD [23, 24].

Sortilin has been linked with circulating low-density lipoprotein cholesterol (LDL-C) levels and with the risk of developing atherosclerosis [25–27]. Sortilin also appears to be a key factor in hepatic and muscular response to insulin, suggesting that it could be a link between insulin resistance and hypercholesterolemia. A study demonstrates that sortilin correlates with the incidence of major adverse cardiovascular events in T2DM [22].

Lipid profiles show the potential markers that can be used in predicting glycemic control in patients with T2DM [28].

This study aimed to examine the effect of oral FAS for 12 weeks on levels of serum Hcy and sortilin, glycemic control, lipid profile and insulin resistance in patients with T2DM. The primary outcome was the glycemic control [fasting blood glucose, glycated hemoglobin, and homeostasis model assessment-insulin resistance (HOMA-IR)]. The secondary outcome was the changes in serum levels of Homocysteine, sortilin, and lipid profile.

## Patients and method

### Study design

This was a randomized controlled double-blind clinical trial that was performed on 100 patients with T2DM aged between 45 and 75 years, who attended the Damanhour National Medical Institute, Egypt during the period starting from April 2020 to February 2021. The study protocol was approved by the Research Ethics Committee of the Faculty of Pharmacy, Damanhour University (Ref No. 122PP48) and adhered to the principles of the Declaration of Helsinki regarding the ethical principles for research involving human subjects. Written informed consent was obtained from all the patients prior to participation in the study.

Patients who visited the outpatient clinic of Internal Medicine and Diabetes Department were evaluated for their eligibility in the present study. Inclusion criteria were patients with a confirmed diagnosis of T2DM at the time of admission under metformin treatment (1500 mg daily for more than six months), other medications, and diet therapy continued unchanged during the study period.

The patients having the following circumstances were excluded from this study: thyroid dysfunction, hepatic failure, renal disorders, cardiovascular disease, gastrointestinal disorders, folate antagonist medication usage (i.e., methotrexate), cigarette smoking (those who smoke greater than or equal to 25 cigarettes per day), pregnancy, and chronic alcohol consumption. The patients who had insulin therapy, consumed folic acid, or treated with statins within the last 3 months were also excluded from the study.

The patients were randomly allocated using computer-generated random sequence in (1:1 ratio) to enroll either in the folic acid group, 50 patients received FAS for 12 weeks (5 mg daily); or in the placebo group, 50 patients received placebo. The sequence was given to an external data manager with no involvement in the study procedures and concealed on a password-protected computer. Once the patient has consented to participate, he assigns the treatment group using the sequentially numbered, opaque, sealed envelope (SNOSE) technique. Both groups were matched for age, weight, glycemic control indicators, and medication use.

The participants were advised to continue their physical activity, routine healthy dietary intake, and medication during the study period.

Adherence to FAS was determined by questioning the participants about the average number of tablets used per week throughout the study period.

Adherence to FAS: Respondents who consumed at least five tablets per week throughout their study duration were classified as adherent, while those who took less than five tablets per week were non-adherent and excluded from the study.

### Blood sampling

Patients attended the hospital in the morning following an overnight fast for 8–12 h. Patients were permitted to sit comfortably for 10 min, and the patient’s height, weight, and blood pressure were assessed. Then venous blood samples (∼10 ml) were collected in ordinary and EDTA vacuum tubes, then centrifuged at 4000 rpm for 10 min at 4 °C.

After blood collection, the samples were instantly cooled, and the serum and the plasma were removed within 1 h and aliquoted into separate tubes, and stored at −80^°^C until analysis.

### Anthropometric evaluation

Anthropometric measurements, age (years), weight (kilograms), and height (centimeters) of the selected patients were evaluated while patients were barefoot and wearing informal dresses. The blood pressure of the patients in the sitting position was measured, following a 15-minute resting period. The following equation was used to calculate body mass index (BMI): weight (kg)/square meter of height (m^2^) [29].

### Biochemical assays

Plasma glucose was assessed by the glucose oxidase method. The ion exchange method using kits obtained from Stanbio Laboratory Company (USA) was used to measure HbA1c%. Serum total cholesterol (TC), high-density lipoprotein cholesterol (HDL-C), and triglycerides (TG) were determined calorimetrically using kits acquired from Elitech Diagnostics Company (France). LDL-C was calculated by the Friedewald formula [30]. Fasting plasma insulin was analysed using Enzyme-Linked Immunosorbent Assay (ELISA) kit (DRG International, Inc., USA). Homeostasis model assessment-insulin resistance (HOMA-IR) was computed according to the following formula: fasting glucose (mg/dl) X fasting insulin (µIU/ml)/405.

hs-CRP was assayed in serum by nephelometric procedure (Dade Behring, BNII, Marburg, Germany).

Circulating serum sortilin and homocysteine were measured by ELISA kits (Cosmo Bio, Carlsbad, CA, USA). The intra and inter-assay coefficients of variation for homocysteine and sortilin, were <10% and <12%, respectively. The sensitivity was estimated to be 0.2 μmol/L-15 μmol/L for homocysteine and 30 pg/ml-2000 pg/ml for sortilin. The serum levels were measured twice, and the results were averaged for each patient.

### Adverse effects

Participants were also followed-up by telephone calls and through direct meetings during the study to assess their adherence and report any drugs related adverse effects using an adverse effect questionnaire. The adverse effects were also collected from the patients’ laboratory data and patient sheets.

### Statistical analyses

The sample size was calculated using G*Power software version 3.1.9.7 (Institut für Experimentelle Psychologie, Heinrich Heine Universität, Dfüsseldorf, Germany). It was estimated that a total sample size of 100 patients would have a power of 97% to detect a medium to large effect size of 0.80 in the measured parameters. Data were tested for normality by Kolmogorov–Smirnov test. Chi–squared or Fisher’s exact test (2-sided) was used to compare qualitative data which were described as numbers and percentages. Continuous data were expressed as means ± SD and paired *t*-test was used to determine differences in mean anthropometric and clinical characteristics before and after FAS. Independent sample *t*-test was used to compare between groups. Pearson’s correlation test was accomplished to evaluate the relationship between changes in Hcy, sortilin, and other variables after supplementation. Receiver-operating characteristic (ROC) area under the curve (AUC) analysis was used to evaluate the sensitivity of the measured variables among T2D patients. Furthermore, multiple regression model was used to determine whether there was any association between HOMA-IR with Hcy, hs-CRP, and sortilin. All statistical analyses were performed using the SPSS for Windows ver. 23.0 software. The reported adverse events in both groups were expressed as numbers and percentage. The level of significance set for the study was *P* < 0.05.

## Results

**Fig. 1:**
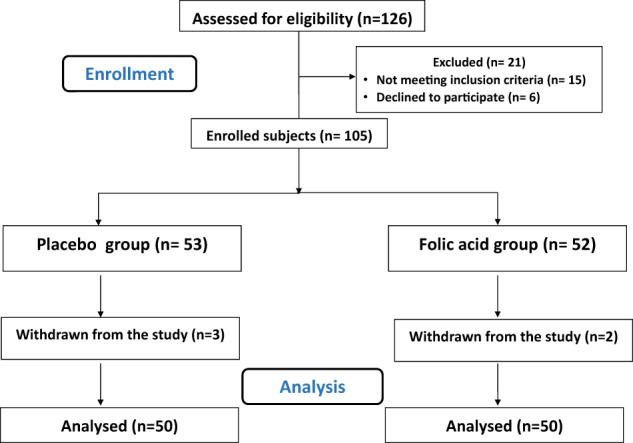
CONSORT flowchart of the study.

At first, 105 patients participated in this study after verifying the inclusion and exclusion criteria as previously stated for patients’ selection. Although, 5 patients were excluded from the study throughout 3-month treatment period for the following purposes: two patients were non-adherent to folic acid administration and 3 patients were non-adherent to their medications. Finally, 100 patients were continued in this study (Fig. 1).

### Baseline and anthropometric characteristics of the participants in both groups

The folic acid group showed no significant difference in baseline characteristics compared to the placebo group (*P* > 0.05). This study included 45 males. Participants’ mean age was 54.46 ± 7.81 *vs* 52.86 ± 9.46 years in the placebo and folic acid groups, respectively. The mean duration of diabetic status was 7.14 ± 3.76 *vs* 7.82 ± 3.94 years in the placebo and folic acid groups, respectively. Associated diseases recorded for the participants were HTN (54% vs 46%) and hyperlipidemia (28% vs 24%) in the placebo and folic acid groups, respectively. Among the participants, 34% were smokers in the placebo group whereas 30% were smokers in the folic acid group with in-significant differences between both groups. Commonly co-administered medications were ACEIs (38% vs 46%), ARBs (34% *vs* 42%), Beta-blockers (14% vs 6%) and CCB (20% vs 22%) in placebo and folic acid group, respectively. Both groups demonstrated no statistically significant change in demographic data, medical history, and medication history (Table 1).

**Table 1:** General characteristics of the study participants at baseline.

Variables	Placebo group (*n* = 50)	Folic acid group (*n* = 50)	*P*-value
Male, *n* (%)	23 (46)	22 (44)	0.841
Age (years)	54.46 ± 7.81	52.86 ± 9.46	0.36
Duration of diabetes (years)	7.14 ± 3.76	7.82 ± 3.94	0.38
Hypertension, *n* (%)	27 (54)	23 (46)	0.424
Hyperlipidemia, *n* (%)	14 (28)	12 (24)	0.648
Smoker, *n* (%)	17 (34)	15 (30)	0.668
ACEIs use, *n* (%)	19 (38)	23 (46)	0.418
ARB use, *n* (%)	17 (34)	21 (42)	0.410
BB use, *n* (%)	7 (14)	3 (6)	0.182
CCB use, *n* (%)	10 (20)	11 (22)	0.806
**OHA Co-treatment with Metformin**			
Glimepiride, *n* (%)	20 (50)	25 (50)	0.315
Glibenclamide, *n* (%)	18 (36)	15 (30)	0.523
Vildagliptin, *n* (%)	12 (24)	10 (20)	0.629

Data presented as number (percentage) or mean ± SD.

*ACEIs* Angiotensin-converting enzyme inhibitors, *ARBs* Angiotensin Receptor Blockers, *BB* Beta-blockers, *CCB* Calcium channel blocker, *OHA* Oral hypoglycemic agents.

Data analysed using independent sample *t*-test or Chi-square as appropriate. Statistically significant between groups at *P* < 0.05.

### Glycemic control, HOMA-IR, and body mass index

The mean BMI of the participants not changed in studied groups after folic acid supplementation (*P* > 0.05). FAS for 3 months reduced fasting blood glucose (FBG) (*P* = 0.0005), HbA1c (*P* = 0.0002), insulin level (*P* < *0.0001)* and HOMA-IR *(P* < *0.0001)*. After receiving FAS for 3 months, placebo group showed no statistically significant differences in FBG, HbA1c, insulin level, and HOMA-IR *(P* > 0.05*)*. After 3 months of intervention, a significant change was found between both groups regarding FBG (*P* = 0.001), HbA1c (*P* = 0.003), and HOMA-IR *(P* = 0.004*)*. On the other hand, insulin levels didn’t change significantly in both groups (Table 2).

**Table 2:** Comparison of biochemical parameters at baseline and after folic acid supplementation between two study groups.

Variables		Placebo group (*n* = 50)	Folic acid group (*n* = 50)	*P-value
BMI (kg/m^2^)	Baseline	27.69 ± 2.64	28.34 ± 3.11	0.265
	After 3 months	27.44 ± 3.01	27.78 ± 3.30	0.584
	‡*P*-value	0.06	0.35	
FBG (mg/dl)	Baseline	120.30 ± 17.63	117.86 ± 16.77	0.48
	After 3 months	116.58 ± 14.13	107.60 ± 10.90	0.001
	‡*P*-value	0.108	0.0005	
HbA1c (%)	Baseline	7.56 ± 0.76	7.57 ± 0.79	0.965
	After 3 months	7.41 ± 0.85	6.95 ± 0.62	0.003
	‡*P*-value	0.09	0.0002	
Insulin (µIU/ml)	Baseline	18.98 ± 5.47	19.68 ± 5.06	0.238
	After 3 months	18.45 ± 5.44	16.98 ± 4.68	0.151
	‡*P*-value	0.927	<0.0001	
HOMA-IR	Baseline	5.50 ± 1.97	5.70 ± 1.58	0.590
	After 3 months	5.30 ± 1.68	4.46 ± 1.09	0.004
	‡*P*-value	0.131	<0.0001	
TG mg/dl	Baseline	142.22 ± 15.24	148.81 ± 31.34	0.185
	After 3 months	131.06 ± 15.89	127.92 ± 16.87	0.340
	‡*P*-value	0.0005	<0.0001	
TC mg/dl	Baseline	175.40 ± 22.44	177.98 ± 24.19	0.582
	After 3 months	159.66 ± 20.25	159.66 ± 20.25	0.354
	‡*P*-value	<0.001	<0.0001	
LDL-C mg/dl	Baseline	112.65 ± 23.64	113.60 ± 26.89	0.851
	After 3 months	95.56 ± 18.12	99.42 ± 20.73	0.324
	‡*P*-value	<0.001	<0.001	
HDL-C mg/dl	Baseline	34.31 ± 2.38	34.62 ± 2.41	0.521
	After 3 months	34.35 ± 2.37	34.65 ± 2.43	0.532
	‡*P*-value	0.568	0.48	
Homocysteine (μmol/L)	Baseline	1.66 ± 0.40	1.74 ± 0.44	0.293
	After 3 months	1.57 ± 0.45	1.25 ± 0.14	0.000
	‡*P*-value	0.124	<0.0001	
Sortillin (ng/ml)	Baseline	0.89 ± 0.28	0.86 ± 0.25	0.559
	After 3 months	0.85 ± 0.35	0.57 ± 0.19	0.000
	‡*P*-value	0.118	<0.0001	
hs-CRP (pg/ml)	Baseline	458.30 ± 50.19	464.69 ± 65.21	0.584
	After 3 months	464.73 ± 52.92	438.05 ± 39.04	0.005
	‡*P*-value	0.089	0.008	

Data presented as mean ± SD.

*BMI* body mass index, *FBG* fasting blood glucose, *HbA1c* glycated hemoglobin, *TG* triglyceride, *TC* total cholesterol, *LDL-C* low-density lipoprotein cholesterol, *HDL-C* high-density lipoprotein cholesterol, *hs-CRP* high-sensitivity C-reactive protein.

‡Paired sample *t*-test statistically significant within groups at *P* < 0.05.

*Independent sample *t*-test statistically significant between groups at *P* < 0.05.

### Lipid profile

After intervention for 3 months, placebo group didn’t show statistically significant differences in TG, TC, LDL-C, and HDL-C levels compared with folic acid group (*P* > 0.05) (Table 2).

### Homocysteine, sortilin, and hs-CRP

**Fig. 2:**
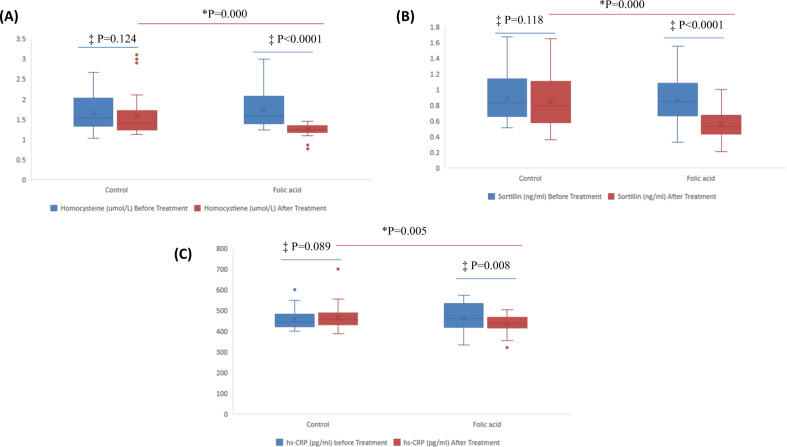
Changes in the measured variables (A) Homocysteine (B) Sortilin (C) hs-CRP. ^‡^Paired sample *t*-test statistically significant within groups at *P*< 0.05. ^*^Independent sample *t*-test statistically significant between groups at *P* < 0.05.

Following FAS for 3 months, serum Hcy levels decreased by 28.2% from 1.74 ± 0.44 μmol/L to 1.25 ± 0.14 μmol/L (*P* < 0.0001). Further, serum sortilin level significantly reduced by 33.7% from 0.86 ± 0.25 ng/ml to 0.57 ± 0.19 ng/ml (*P* < 0.0001). hs-CRP also reduced from 464.69 ± 65.21 to 438.05 ± 39.04 (*P* = 0.008) (Table 2). On the other hand, theses parameters didn’t change significantly in placebo group (Fig. 2).

### Relationship between FBG, Homocysteine, sortilin, and hs-CRP

A positive correlation was found between sortilin with Hcy (*r* = 0.493, *P* = 0.000) and hs-CRP (*r* = 0.237, *P* = 0.018). Further, there was a positive association between Hcy with hs-CRP (*r* = 0.308, *P* = 0.002). A positive association was found between FBG with hs-CRP (*r* = 0.342, *P* = 0.000) (Table 3).

**Table 3:** Association between sortilin, homocysteine, hs-CRP, and FBG after folic acid supplementation.

		Homocystiene (μmol/L)	hs-CRP (pg/ml)	FBG
Sortillin (ng/ml)	*r*	0.493^**^	0.237^*^	0.169
	*P*	0.000	0.018	0.093
Homocystiene (μmol/L)	*r*		0.308^**^	−0.076
	*P*		0.002	0.451
hs-CRP (pg/ml)	*r*			0.342^**^
	*P*			0.000

FBG: fasting blood glucose; hs-CRP, high-sensitivity C-reactive protein.

*Correlation is significant at the 0.05 level (2-tailed).

**Correlation is significant at the 0.01 level (2-tailed).

### Association between HOMA-IR with Hcy, sortilin, hs-CRP, and insulin levels via multivariate regression analysis

Multiple linear regression analysis was conducted to find whether there was an independent correlation between HOMA-IR with Hcy, sortilin, hs-CRP, and insulin levels. The analysis results demonstrated that HOMA-IR levels were inversely and independently correlated with Hcy, whereas HOMA-IR were positively related to sortilin, hs-CRP and insulin levels (Table 4).

**Table 4:** Multiple linear regression assessment of associated parameters with HOMA-IR in the studied participants (Adjusted *R*2 = 0.852).

Model	Unstandardized coefficients		Standardized coefficients	*t*	*P*-value	95.0% Confidence interval for B	
	β	Std. error	β			Lower Bound	Upper Bound
(Constant)	−1.259	0.602		−2.090	0.039	−2.455	−0.063
Sortillin (ng/ml) After Treatment	0.447	0.213	0.095	2.096	0.039	0.024	0.871
Homocystiene (µmol/L) After Treatment	−0.521	0.180	−0.133	−2.903	0.005	−0.878	−0.165
Insulin-A (µIU/ml) After Treatment	0.266	0.011	0.922	23.305	0.000	0.243	0.288
hs-CRP (pg/ml) After Treatment	0.004	0.001	0.134	3.237	0.002	0.002	0.007

Dependent variable: HOMA-IR after multiple linear regression analysis was used. β: Unstandardized regression coefficient.

*CI* confidence interval, *HOMA-IR* homeostasis model assessment of insulin resistance, *hs-CRP* high-sensitivity C-reactive protein.

*P < 0.05 was significant.

### Area under ROC curve of different measured parameters in the studied groups

**Fig. 3:**
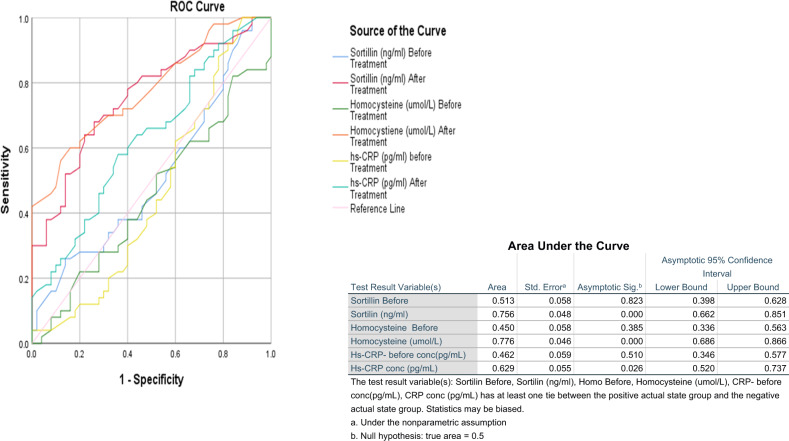
Area under ROC curve of the determined parameters.

Figure 3 shows ROC-AUC of biomarkers among diabetic patients in the both groups before and after intervention duration. Hcy was the most sensitive (AUC = 0.776, *P* = 0.000) followed by sortilin (AUC = 0.756, *P* = 0.000) whereas hs-CRP level was the lowest AUC (0.629, *P* = 0.026) after 3 months of intervention.

### Reported adverse events

The most prevalent side effects were nausea, loss of appetite (14% *vs* 8%), bloating, gas, stomach pain (12% *vs* 2%), unpleasant taste (10% *vs* 2%), and confusion (6% *vs* 0%) in folic acid and placebo group respectively with insignificant difference between both groups as shown in Table 5.

**Table 5:** Reported adverse events in both groups.

	Placebo group (*n* = 50)		Folic acid group (*n* = 50)		*P-*value
	No.	%	No.	%	
Nausea, loss of appetite	4	8	7	14	0.338
Bloating, gas, and stomach pain	1	2	6	12	0.050
Unpleasant taste	1	2	5	10	0.092
Confusion	0	0	3	6	0.079

Data are presented as number and percent.

Data were analyzed by Chi–square test.

Significance level was set at *P* < 0.05.

## Discussion

In the present study, the folic acid impacts as co-treatment in diabetic patients on circulating levels of Hcy, sortilin, indices of glycemic control, and insulin resistance and lipid profile were investigated.

Our results revealed that treatment with folic acid (5 mg/d) for 12 weeks in diabetic patients receiving metformin treatment significantly improved serum levels of FBG, HbA1c, HOMA-IR, Hcy, sortilin, and hs-CRP.

Several studies indicated that blood Hcy levels are inversely correlated to plasma levels of folic acid [31, 32].

Our results showed that FAS reduced Hcy level by 28.2%, which correspond with other research’s findings; although, the percentage drop varies [33–35]. These discrepancies could be explained by changes in treatment duration and folic acid dose [7].

There is a substantial evidence that FAS may improve insulin resistance [36]. Our findings estimated that FAS decreased the level of insulin and HOMA-IR, which is in accordance with previous studies. A study confirmed that plasma insulin level and HOMA-IR were significantly decreased in overweight individuals after treatment with folic acid (2.5 mg/d) for 3 months [37]. Another study by Gargari et al. showed that FAS (5 mg/d) for eight weeks in obese patients with T2DM improved glycemic control and insulin resistance [10].

Several mechanisms have been proposed for the involvement of FAS and Hcy reduction in the pathogenesis of insulin resistance and T2DM. A potential mechanism is that Hcy may block insulin-stimulated tyrosine phosphorylation of insulin receptor β-subunit and its substrates, resulting in suppression of glycogen production [38]. Subsequently resulting in insulin resistance and hyperglycemia. Another suggested explanation is that folic acid improves endothelial dysfunction induced by high Hcy by preventing nitric oxide synthase dysfunction, which may be beneficial to glycomtabolism [7]. Folic acid has also been reported to diminish Hcy, which in turn reduces oxidative stress and systemic inflammation, which can disrupt insulin signaling and diminish release of insulin from pancreatic β cell [39].

Our results showed that FAS has resulted in significant reduction in FBG and HbA1c levels, which are in consistence with a meta-analysis [40] that reported a link between a decrease in Hcy level and a decrease in fasting glucose and HbA1c levels, indicating the role of Hcy as an intermediary. The findings might indicate that a significant reduction in Hcy is required for improvement of glycemic control, which might be accomplished efficiently by better compliance and/or increase of FAS dose.

In consistence with our results, Chiarelli et al. revealed an elevated plasma Hcy level in adolescents and young adults with type 1 DM and microvascular consequences; they also reported a positive link between plasma Hcy level and HbA1c [41].

Inconsistence with our results, other studies showed that FAS didn’t decrease HbA1c significantly in T2DM patients [10, 42].

Hyperhomocysteinemia tends to be a greater (1.9-fold) predictor for death in patients with T2DM than in nondiabetic individuals [43]; a meta-analysis demonstrated that FAS can diminish Hcy in patients without cardiovascular disease history. FAS could reduce the risk of cardiovascular events in T2DM patients, particularly as a primary preventative strategy [3].

Our results showed a significant decrease in hs-CRP level in diabetic patients after FAS, which are in consistence with a study [37] that estimated reduction of some inflammatory mediators levels as hs-CRP regardless of weight change after FAS, implying that FAS could have a therapeutic effect in the atherogenesis and cardiovascular diseases prevention.

In another study, patients with acute ischemic stroke who received a combination of 5 mg of folic acid with vitamin B complex for 2 weeks showed significant reduction in hs-CRP levels [44].

According to a meta-analysis, T2DM patients had a greater decrease in hs-CRP levels following FAS compared with healthy controls. T2DM patients are thought to have greater or equivalent levels of inflammatory biomarkers compared to healthy individuals [45].

Folic acid supplementation reduces hs-CRP concentration through several mechanisms. The reduction in Hcy levels following FAS has been linked to a reduction in oxidative stress [46]. Furthermore, the reduction in insulin resistance after FAS may result in reduced synthesis of inflammatory factors [47]. Other researchers, on the other hand, claim that FAS has no effect on serum level of CRP [48].

Our results revealed that there were no significant changes in levels of TC, TG, LDL-C, and HDL-C following FAS. In agreement with a pervious study by Tabrizi et. al., 2018 which concluded that Folate supplementation did not affect blood pressures and lipid profiles among patients with metabolic diseases [49].

Gargari et. al., found no significant differences in lipid profile following 8 weeks of FAS (5 mg/d) in obese males with T2DM, which is consistent with earlier studies [7]. Similarly, Mangoni et al. reported that FAS with 5 mg/d for 4 weeks did not induce significant differences in levels of TC, TG, LDL-C, and HDL-C in T2DM patients [42].

Our results were incompatible with a trial on post-menopausal Korean females with T2DM that found a decrease in level of LDL-C along with decrease in LDL-C/HDL-C and TC/HDL-C ratios after FAS with 800 μg/d for 8 weeks [9].

An experimental animal study demonstrated a correlation between higher levels of Hcy as well as reduced HDL-C and elevated levels of TC and non-HDL-C [50]. Elevated levels of Hcy have also been demonstrated to decrease the enzymatic activities of two HDL- metabolizing enzymes [51]. Increased Hcy levels were shown to increase LDL receptors expression and LDL-C transport [52]. As a result, improved lipid profile caused by FAS-induced decline in serum levels of Hcy.

Sortilin has been linked to many pathological processes including inflammation and calcification of arterial wall, insulin resistance, and disrupted lipoprotein metabolism [24, 53].

The present study showed that levels of hs-CRP were significantly greater at baseline assessment of studied patients. Furthermore, among the studied patients, the levels of hs-CRP and sortilin had a substantial positive correlation. Similarly, plasma sortilin has demonstrated significant association with hs-CRP, which has been found to be formed in vascular smooth muscle cells, in patients with coronary artery disease whose platelets were suppressed by aspirin treatment [25]. In another study, newly diagnosed T2DM patients had higher levels of hs-CRP than controls, despite no correlation between levels of hs-CRP and sortilin [23]. Therefore, hs-CRP is a promising predictor for atherosclerotic cardiovascular disorders in T2DM patients [54].

Conflicting data were reported in clinical trials regarding the association between sortilin and lipids.

To our knowledge, this is the first study to investigate the association between sortilin and lipid profile in Egyptian patients with T2DM. In the current study, we found no link between sortilin and lipid profile. Consistently with the present results, Oh et. al., reported insignificant correlation between levels of sortilin and lipid profile [21].

Inconsistently, Ogawa et al. showed that there was a significant association between sortilin levels and LDL-C as well as TC [25]. In addition, it was shown in a preclinical trial that in sortilin-deficient mice, the lipoproteins production from the liver and hypercholesterolemia were decreased [55]. Likewise, mice lacking macrophage sortilin illustrated decreased atherosclerotic plaques due to LDL uptake reduction [56]. Contrary, Demir et al. reported a negative correlation between sortilin levels and TC, LDL-C and TG, while levels of sortilin were positively linked with HDL-C in newly diagnosed T2DM patients suggesting that sortilin may contribute to dyslipidemia in T2DM. Another study showed that hepatic sortilin decreases hepatic apolipoprotein B production while enhancing LDL-C degradation [57].

This study has several limitations: including small sample size, short period of follow-up, and the lack of intention to treat analysis. Further large-scale studies are needed, and further extension of this work is warranted.

## Conclusion

FAS might be beneficial for reducing Hcy and sortilin levels, improving glycemic control and insulin resistance in T2DM patients. As a result, FAS could have a possible contribution in the primary prevention of cardiovascular events in diabetic patients. Homocysteine and sortilin could be used as therapeutic targets for T2DM treatment and could aid clinicians for identification of cardiovascular risk in diabetic patients.

## Data Availability

The data that support the findings of this study are available upon reasonable request.
